# Gut Microbiome-Driven Microglial Activation Links Dysbiosis to Pain in Interstitial Cystitis/Bladder Pain Syndrome

**DOI:** 10.21203/rs.3.rs-9087060/v1

**Published:** 2026-04-23

**Authors:** Shivesh Ghura, Habib Jmii, James Griffith, Anthony J. Schaeffer, David J. Klumpp

**Affiliations:** Northwestern University; Northwestern University; University of Chicago Medicine and Biological Sciences; Northwestern University; Northwestern University

**Keywords:** Interstitial Cystitis, Bladder Pain Syndrome, Pelvic Pain, Gut microbiome, Microglia, Neuroinflammation, Genitourinary Pain Index (GUPI)

## Abstract

**Background:**

Interstitial cystitis/bladder pain syndrome (IC/BPS) is a debilitating condition of chronic pelvic pain associated with urinary frequency and comorbid anxiety and depression. Recent studies in IC/BPS patients and rodent models implicate fecal dysbiosis and increased systemic exposure to endotoxin. These changes potentially elicit innate immune responses via the activation of microglial cells in the central nervous system, key mediators of pain. Microglial ablation and inactivation have previously been associated with analgesia in preclinical studies, underscoring the role of microglia in IC/BPS pain. Here, we investigated whether IC/BPS-associated fecal microbiota differentially activate microglia and whether activation correlates with patient symptoms.

**Methods:**

Microbiome-microglia interactions were assessed using three complementary *in vitro* culture models: BV2 cells, enriched primary microglia (~ 95% microglia), and mixed glial cultures (microglia and astrocytes). Microglial cultures were exposed to heat-killed, stool-derived microbiota, and the pro-inflammatory cytokines tumor necrosis factor-α (TNF-α), RANTES/CCL5, and interleukin-6 (IL-6) were quantified by ELISA. Cytokine levels were evaluated for patients and controls and correlated with patient-reported genitourinary pain index (GUPI) scores.

**Results:**

In all culture models, microglia exhibited significantly increased proinflammatory responses to fecal microbiota of IC/BPS patients relative to controls. Mixed glial cultures, incorporating astrocyte-microglia interactions, exhibited the most robust cytokine responses. Cytokine levels positively correlated with GUPI pain scores.

**Conclusions:**

Together, these findings further support a role for gut dysbiosis in IC/BPS symptoms and suggest microglial activation and glial-glial interactions as a contributing mechanism. Understanding gut-brain axis interactions in IC/BPS will thus enable development of novel microbiome-based therapies for treating IC/BPS patients.

## Background

Urologic chronic pelvic pain syndrome (UCPPS) is an umbrella term encompassing chronic visceral pain conditions, including interstitial cystitis/bladder pain syndrome (IC/BPS). IC/BPS is a debilitating disorder affecting women predominantly and is characterized by chronic pelvic pain and urinary frequency and urgency[1-7]. IC/BPS is also associated with anxiety and depression as well as gut microbiome alterations[5]. Current treatment options for IC/BPS focus on symptoms and are non-curative, reflecting an incomplete mechanistic understanding of disease pathogenesis and a lack of validated biomarkers.

Neuroimaging studies from the MAPP Research Network have revealed changes in white matter integrity and accelerated brain aging that correlate with pain severity[8-12]. These findings indicate intrinsic CNS remodeling in IC/BPS and underscore the need to identify specific biological pathways linking peripheral pathology to central pain processing. An emerging hypothesis posits that initiating events in the bladder lead to neuronal sensitization, which is subsequently amplified by immune-mediated mechanisms involving central nervous system (CNS) immune cells, including microglia[7]. However, the pathways linking peripheral pathology to central pain processing in IC/BPS remain poorly defined.

Multiple studies in IC/BPS patients have reported gut dysbiosis, including depletion of bacterial taxa involved in the biosynthesis of key metabolites known to modulate gut health and gut-brain interactions[5, 13, 14]. Preclinical models further support a role for gut microbiota in IC/BPS pathophysiology, where mice lacking acyloxyacyl hydrolase (AOAH) develop chronic pelvic pain resembling IC/BPS with associated gut dysbiosis, where fecal transplant reduces pain[15, 16]. Notably, AOAH-deficient mice display increased intestinal permeability and elevated circulating endotoxin levels, which may cross the blood-brain barrier and activate immune CNS pathways[15]. To evaluate the clinical relevance of such interactions, we examined responses of microglia to patient stool microbiota. Microglia are macrophage-like CNS innate immune cells with central roles in pain. Importantly, microglia express Toll-like receptors and receptors for gut metabolites and thus are also transducers of gut-brain interactions. Here, we report that stool microbiota of IC/BPS patients elicit stronger pro-inflammatory cytokine responses in cultured microglia, relative to controls, and that cytokine responses correlate with patient pain scores. These findings bolster a role for gut-brain interactions in IC/BPS mediated by microglia and point to novel therapeutic opportunities.

## Results

### IC/BPS patients and healthy controls were recruited.

IC/BPS patients (n = 16) and healthy controls (n = 16) were recruited through the Urology Clinic using established inclusion and exclusion criteria[5]. IC/BPS participants were all female and exhibited significantly higher GUPI pain, urinary, quality of life, and total scores compared to controls (Table 1). Although the healthy control cohort was more racially diverse and included a higher proportion of Black/African American participants, race and ethnicity were not significantly associated with disease status in this cohort (Table 1). However, induced microglial cytokine responses did not differ among racial and ethnic groups of control participants (Additional File 1), suggesting that any differences in responses to patient and control microbiota were not influenced significantly by racial and ethnic differences within the participant cohorts.

### IC/BPS stool microbiota elicit increased microglial proinflammatory cytokine secretion.

We previously demonstrated that exposure of BV2 microglial cultures to heat-killed stool slurry derived from IC/BPS patients induces higher expression of the microglial activation marker CD68 compared to slurry from healthy controls, suggesting that IC/BPS-associated microbiota can directly or indirectly activate microglia[15]. Here, we extended these findings to assess cytokine and chemokine secretion in complementary cell models using the mouse BV2 cell line, enriched primary cortical murine microglia, and mixed cultures of primary cortical microglia and astrocytes to mimic cell-cell interactions in the CNS. To validate the cellular composition and purity of each *in vitro* system, immunofluorescence staining and flow cytometric analyses were performed, confirming appropriate enrichment of microglial populations and astrocyte representation in mixed glial cultures (Additional File 2). Primary microglia were approximately 91% CD11b+, consistent with successful enrichment of microglia, whereas mixed glial cultures were approximately 15% CD11b+, approximating the physiological proportion of microglia in the murine brain. Initial multiplex cytokine profiling in enriched primary microglia identified TNF-α, CCL5, and IL-6 as consistently responsive inflammatory mediators among a broader panel of analytes, guiding focused downstream analyses (Additional File 3).

**Figure 1:**
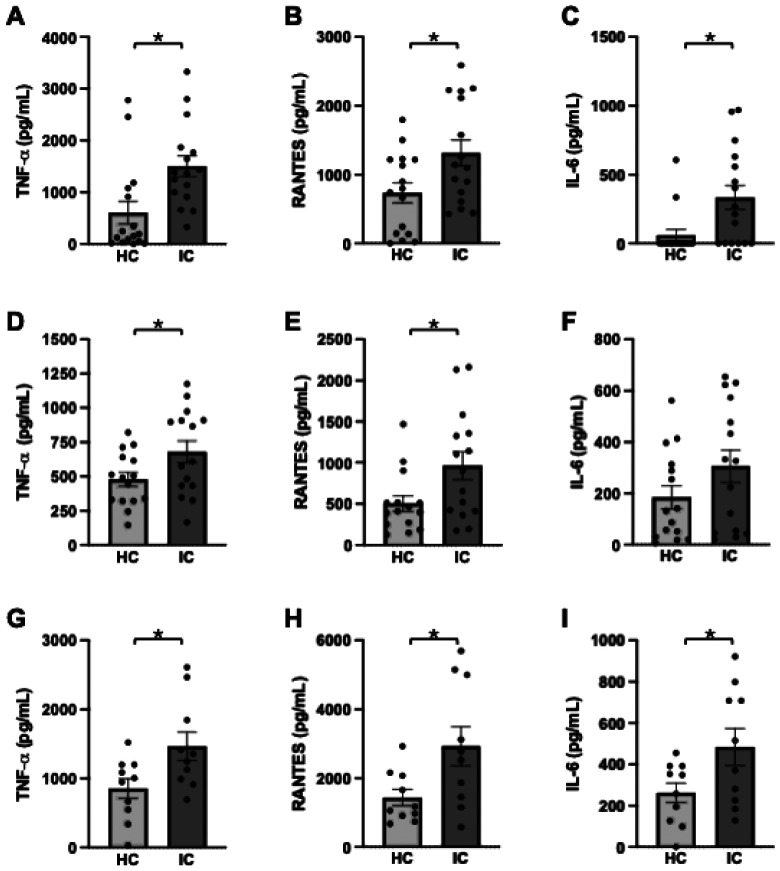
IC/BPS stool microbiota elicit increased proinflammatory cytokine secretion across microglial culture systems. BV2 cells (a–c), enriched primary microglia (d–f), and mixed glial cultures (g–i) were treated with 1 μg/mL heat-killed stool slurry derived from healthy controls (HC) or IC/BPS patients (IC). After stimulation, concentrations of TNF-α (a, d, g), RANTES (b, e, h), and IL-6 (c, f, i) in culture supernatants were quantified by ELISA. Data are presented as mean ± SEM, with individual data points representing independent patient stool samples. Statistical comparisons between HC and IC groups were performed using two-tailed unpaired Student’s t-tests. p < 0.05 indicates statistical significance.

Microglial cultures exposed to heat-killed stool slurry derived from IC/BPS patients or healthy controls, and culture supernatants were evaluated by ELISA for TNF, RANTES, and IL-6 (Fig. 1). In BV2 microglial cultures, IC/BPS stool slurries elicited a greater increase in tumor necrosis factor-α (TNF-α), CCL5/RANTES, and interleukin-6 (IL-6) secretion relative to healthy control microbiota (Fig. 1A-C). Similar patterns were observed in enriched primary microglia, where exposure to IC/BPS stool resulted in significantly elevated TNF-α and CCL5 secretion, although IL-6 showed a more modest and variable response (Fig. 1D-F). In mixed glial cultures that incorporate astrocyte-microglia interactions, IC/BPS stool induced significantly higher secretion of TNF-α, CCL5, and IL-6 compared to healthy controls, with overall cytokine levels exceeding those observed in microglia-only cultures (Fig. 1G-I, compared to D-F). Comparative analyses further demonstrated strong concordance in cytokine responses between enriched microglia and mixed glial cultures following IC/BPS stool exposure, supporting the robustness and reproducibility of these inflammatory signatures across in vitro systems (Additional File 4).

### Microglial cytokine responses to IC/BPS microbiota correlate with patient-reported pain.

**Figure 2:**
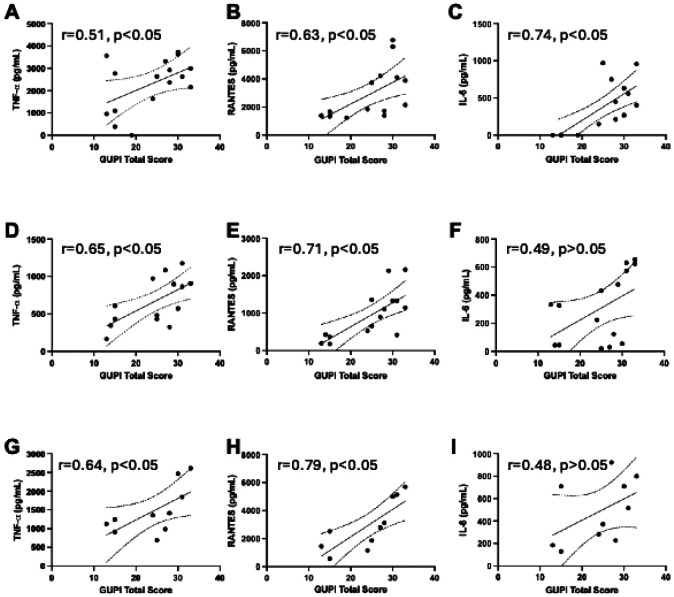
Microglial cytokine responses to IC/BPS stool microbiota correlate with patient-reported pain severity. Cytokine levels secreted by BV2 microglial cell line (a–c), enriched primary microglia (d–f), and mixed glial cultures (g–i) in response to patient-derived stool slurry were plotted against the corresponding total Genitourinary Pain Index (GUPI) score of the same patient. TNF-α (a, d, g), RANTES (b, e, h), and IL-6 (c, f, i) concentrations were quantified by ELISA. Correlations were assessed using two-tailed Pearson’s correlation analysis; correlation coefficients (*r*) and *p* values are shown in each panel. Solid lines indicate linear regression fits with dashed lines representing 95% confidence intervals.

To assess the clinical relevance of microbiome-induced glial activation, we next examined whether cytokine responses correlated with patient-reported pain severity, as measured by the Genitourinary Pain Index (GUPI). Across all *in vitro* systems, increased cytokine secretion in response to IC/BPS stool microbiota was positively associated with higher GUPI scores (Fig. 2). In BV2 cultures, secretion of TNF-α, CCL5, and IL-6 demonstrated moderate to strong positive correlations with patient-reported pain severity (Fig. 2A-C). Similar relationships were observed in enriched primary microglia, where TNF-α and CCL5 levels correlated significantly with GUPI scores, while IL-6 showed a weaker, non-significant association (Fig. 2D-F). In mixed glial cultures, cytokine secretion exhibited the strongest correlations with pain severity, with TNF-α, CCL5, and IL-6 all demonstrating significant positive relationships with GUPI scores (Fig. 2G-I). Together, these findings link patient-derived IC/BPS microbiota to microglial and glial inflammatory responses that scale with clinical pain severity, supporting a translational association between gut dysbiosis, central immune activation, and IC/BPS symptom burden.

## Methods

### Human subjects and stool sample collection.

Patient samples were collected under Protocol STU00055668 approved by the Institutional Review Board of Northwestern University (ClinicalTrials.gov ID NCT01738464). IC/BPS patients and healthy controls were recruited through the Urology Clinic and enrolled using standard pelvic pain inclusion and exclusion criteria, as previously described[5, 17]. Cohort demographics, including race and ethnicity distribution, are summarized in Table 1. Because Black/African American participants were overrepresented in the healthy control cohort, we additionally examined cytokine distributions stratified by ethnicity (Additional File 1).

Symptom severity was assessed using the Genitourinary Pain Index (GUPI) questionnaire[18] administered at the time of enrollment (Table 1). Stool samples were collected according to approved protocols adapted from the Human Microbiome Project Manual of Procedures, as previously described[5]. Briefly, participants collected stool samples at home, froze them immediately, and shipped samples on wet ice. Upon receipt, samples were aliquoted and stored at −80°C until further processing.

### Preparation of stool slurry.

Frozen stool samples were weighed and resuspended in 1 mL Dulbecco’s phosphate-buffered saline. Samples were vortexed, heat-inactivated at 65°C for 15 min, filtered through a 0.22 μm filter, and applied to cultures at a final concentration of 1 μg/mL.

### Microglia culture models and stimulation.

Immortalized BV2 murine microglial cells (AcceGen, Cat# ABC-TC212S) were maintained in Dulbecco’s Modified Eagle Medium supplemented with 10% fetal bovine serum (FBS) and 1% penicillin-streptomycin. BV2 cells were plated at 1 x 10^5^ cells/well, and on reaching confluency were subsequently treated with stool slurry for 6 h prior to supernatant collection.

C57BL/6J mice were bred in facilities of Northwestern's Center for Comparative Medicine and maintained on a 12hr:12hr light:dark cycle, and all procedures were performed under an approved protocol of the Institutional Animal Care and Use Committee of Northwestern University (IS00026133). Primary mixed glial cultures (~ 80–90% astrocytes, ~ 10–20% microglia) were prepared from postnatal day 0–2 mouse cortices. Briefly, neonatal pups were anesthetized by chilling on ice and then quickly decapitated with sharp, sterilized scissors. Cortices were dissected, meninges removed, and tissue enzymatically dissociated using 0.25% trypsin and 1 U/μL DNase I for 25 min at 37°C. The resulting cell suspension was passed through a 40 μm cell strainer and plated in glial maintenance medium consisting of DMEM supplemented with 10% FBS, 1% penicillin-streptomycin, and 2.5% GlutaMAX. Mixed glial cultures were maintained until confluency and used for experiments between days i*n vitro* (DIV) 10–12. Cells were trypsinized and replated into 24-well plates at a density of 1.4 x 10^6^ cells per plate prior to treatment with stool slurry for 24 h.

Enriched microglial cultures were obtained using a modified shake-off protocol[19]. Mixed glial cultures were grown to confluency until microglia were abundantly present (DIV 13–15). Cultures were incubated with Versene (0.12 mM EDTA) at 37°C for 2–3 min, followed by orbital shaking at 250 rpm for 3.5-4 h at 37°C in fresh medium. The supernatant containing detached microglia was collected, pelleted, and seeded at a density of 0.5 x 10^5^ cells per well onto poly-L-lysine coated 24-well plates. Enriched microglia were maintained in glial maintenance medium supplemented with 10 ng/mL recombinant murine macrophage colony-stimulating factor and treated with stool slurry for 24 h.

### Cytokine measurements.

Cell culture supernatants were collected following treatment and analyzed for cytokine secretion using enzyme-linked immunosorbent assay (ELISA). Mouse TNF-α, CCL5/RANTES, and IL-6 DuoSet ELISA kits (R&D Systems) were used according to the manufacturer’s instructions. Cytokine concentrations were calculated by four-parameter logistic curve fitting using GraphPad Prism software. For multiplex cytokine profiling, the Mouse Cytokine Array C3 (RayBiotech) was used according to the manufacturer’s protocol.

### Immunocytochemistry.

Immunofluorescence staining was performed on BV2 cells, enriched primary microglial, and mixed glial cultures. Briefly, cells were washed with PBS and fixed in 4% paraformaldehyde (PFA, Sigma-Aldrich) for 20 min. After washing, cells were permeabilized and blocked in PBS containing 0.2% bovine serum albumin and 0.1% Triton X-100 for 60 min. Cultures were incubated overnight at 4°C with primary antibodies against mouse Iba1 (1:200; Cat# 019-19741, Fujifilm) and rabbit GFAP (1:500; Cat# 3670S, Cell Signaling Technology). Following washes, cells were incubated with appropriate fluorescent secondary antibodies (1:200, Invitrogen Life Technologies) and DAPI (1:1000, Invitrogen Life Technologies) for 30 min at room temperature. Images were acquired using a Nikon Ti2 widefield microscope (Nikon).

### Flow cytometry.

Cells were harvested, pelleted, and resuspended in anti-mouse CD16/CD32 Fc blocking solution prior to staining. Samples were blocked on ice for 30 min and stained with fluorophore-conjugated antibodies against surface markers P2Y12 (anti-P2RY12 PE, Cat#848003, BioLegend) and CD11b (anti-CD11b APC, Cat#17-0112-81, Invitrogen Life Sciences) for 1 h on ice. Cells were then fixed in 4% paraformaldehyde for 15 min, permeabilized, and stained for intracellular GFAP (anti-GFAP Alexa488, Cat#53-9892-82, Invitrogen Life Sciences). Samples were resuspended in PBS and analyzed on a FACSymphony A5.2 spectral analyzer. Controls included unstained samples, single-stained controls, and BV2 cells as microglial reference controls.

### Statistical analyses.

Data are presented as mean ± SEM. Comparisons of cytokine secretion between groups were performed using a two-tailed unpaired Student t-test. Correlations between cytokine levels and GUPI scores were assessed using two-tailed Pearson’s correlation test. Normality was assessed using the Shapiro–Wilk test. Statistical analyses were conducted using GraphPad Prism (version 10), and p < 0.05 was considered statistically significant.

## Discussion

IC/BPS is characterized by heterogeneous clinical presentations and poorly defined etiologies, complicating diagnosis, clinical management, and therapeutic development. Identifying convergent biological pathways that link peripheral pathology to central pain processing is a critical unmet need. We previously reported that exposure of BV2 microglia to IC/BPS-associated fecal microbiota induces greater activation compared to healthy control microbiota, as assessed by increased CD68 expression[15]. Here, we extend those findings by demonstrating enhanced secretion of proinflammatory cytokines TNF-α, IL-6, and RANTES/CCL5, in response to patient-derived IC/BPS microbiota (Fig. 1). This effect is consistent across immortalized BV2 cells, enriched primary microglia, and mixed glial cultures. Importantly, the cytokine secretion positively correlates with patient-reported pain severity, as measured by the GUPI scores (Fig. 2). Together, these findings position microglia as a mechanistic node linking gut dysbiosis to CNS immune activation in IC/BPS.

Microglia are central regulators of neuronal excitability and synaptic plasticity during chronic pain states[20]. Prior studies demonstrate that microglial activation contributes to central sensitization through the release of proinflammatory mediators and chemokines, which can directly influence neuronal signaling and glial-neuronal interactions[21, 22]. Microglia also express Toll-like receptors and nuclear receptors for hydrophobic ligands and thus are poised to respond to circulating products of gut microbiota including microbial structural components and metabolites[7]. Consistent with this literature, we observed robust induction of TNF-α, IL-6 and RANTES/CCL5 in response to IC/BPS-associated microbiota across *in vitro* microglial models.

The distinct behavior of IL-6 relative to TNF-α and RANTES in enriched primary microglial cultures likely reflects differences in cytokine regulation and cellular context. IL-6 signaling in the CNS is known to be highly modulatory and is often refined or amplified through astrocyte–microglia interactions, whereas TNF-α and chemokines such as RANTES are rapidly induced as part of early microglia-intrinsic innate responses[23]. Although microglia appear sufficient to mount inflammatory responses to IC/BPS-associated microbiota, our data also highlight the importance of glial crosstalk in shaping CNS inflammation. Mixed glial cultures, which incorporate astrocytes alongside microglia, exhibited amplified cytokine responses compared to enriched microglial cultures, consistent with the known role of astrocytes in propagating and sustaining inflammatory signaling[24, 25]. At the same time, the strong correlation of cytokine responses between enriched microglia and mixed glia supports the use of both systems as complementary and translationally relevant *in vitro* models.

We have previously shown that pharmacological ablation of microglia using the CSF1R inhibitor PLX5622 attenuates pelvic allodynia in the IC/BPS model of AOAH-deficient mice, supporting a functional role for microglia in pain amplification[15]. Similarly, minocycline suppresses microglial activation and has been shown to reduce neuroinflammatory signaling and pain-related outcomes in *in vitro*[26, 27], in animal models of pain[28, 29], and in some clinical studies[30, 31]. Together, these observations reinforce the therapeutic relevance of targeting microglial activation in chronic pain states, including IC/BPS.

Patients with IC/BPS consistently exhibit gut dysbiosis, and symptoms are frequently exacerbated by dietary triggers, supporting a role for gut-derived signaling in pain manifestation[7]. Alterations in fecal microbiota have also been reported in chronic prostatitis/chronic pelvic pain syndrome, suggesting that microbiome-driven mechanisms may extend across UCPPS phenotypes[7, 32].

Accumulating evidence supports a critical role for bidirectional communication between the gut microbiota and the CNS in the pathophysiology of chronic pain, anxiety and depression, and neurodegenerative diseases[33, 34]. In IC/BPS, patients exhibit gut dysbiosis, and symptoms are commonly exacerbated by comestibles including acidic foods and caffeine[35, 36], supporting a role for gut-derived signaling in pain manifestation (reviewed in [7]). Notably, neuroimaging studies from the Multidisciplinary Approach to the Study of Chronic Pelvic Pain (MAPP) Research Network have identified CNS alterations in IC/BPS patients, including changes in white matter microstructure and accelerated brain aging that correlate with pain severity, particularly in women. Although these imaging studies do not directly resolve specific glial populations, white matter alterations and signatures of neuroinflammation are increasingly recognized as reflecting glial dysfunction. Similar imaging patterns suggestive of neuroinflammation have been described across chronic pain conditions, supporting a broader role for glial-mediated CNS remodeling in persistent pain states[37]. Our data begin to bridge this gap by providing a biological pathway through which peripheral dysbiosis may engage CNS immune mechanisms, specifically microglial activation, to influence pain processing.

Collectively, these data support a model in which IC/BPS-associated gut dysbiosis promotes microglial activation, potentially leading to central sensitization and pain amplification. Restoration of microbial homeostasis therefore represents a promising therapeutic strategy. Prior work demonstrating that correction of dysbiosis through fecal microbiota transfer or co-housing alleviates pelvic pain in preclinical models supports the concept that upstream microbial signals are modifiable contributors to CNS dysfunction. Future studies integrating microbiome, metabolomic, and neuroimaging approaches will be critical to determine whether microbiome-targeted interventions, including probiotics or defined microbial consortia, can attenuate microglial activation and reduce pelvic pain in IC/BPS and related UCPPS conditions.

## Conclusions

Patient-derived IC/BPS microbiota elicit robust microglial inflammatory responses that correlate with clinical pain severity. These findings support a mechanistic link between gut dysbiosis and CNS immune activation in IC/BPS and suggest that microbiome-targeted therapies may represent a promising strategy for disease management.

## Supplementary Material

This is a list of supplementary files associated with this preprint. Click to download.

SupplementaryDataGhuraetal.pdf

Additionalfiles.docx

## Figures and Tables

**Table 1:** Clinical cohort characterization of IC/BPS and healthy control donors.

Age	Healthy controls	IC/BPS patients	p-value
	36.31 ± 14.1	41.56 ± 15	0.317
Sex (F, %)	56.25	100.00	0.0028
Genitourinary Pain Index (GUPI)			
GUPI - Pain	0.00	11.94 ± 3.9	< 0.0001
GUPI - Urinary	0.25 ± 0.4	4.25 ± 1.9	< 0.0001
GUPI - QOL	0.12 ± 0.3	7.44 ± 3.2	< 0.0001
GUPI - total	0.37 ± 0.5	23.69 ± 7.5	< 0.0001
Ethnicity, n (%)			
Asian/Asian American	1 (6%)	0 (0%)	0.281
Black/African American	7 (44%)	3 (19%)	
Hispanic/Latino	1 (6%)	2 (13%)	
White	7 (44%)	11 (69%)	
